# Comparison of Hepatitis E Virus Sequences from Humans and Swine, The Netherlands, 1998–2015

**DOI:** 10.3390/v13071265

**Published:** 2021-06-29

**Authors:** Boris M. Hogema, Renate W. Hakze-van der Honing, Michel Molier, Hans L. Zaaijer, Wim H. M. van der Poel

**Affiliations:** 1 Sanquin Blood Supply Foundation, 1066 CX Amsterdam, The Netherlands; B.Hogema@sanquin.nl (B.M.H.); m.molier@sanquin.nl (M.M.); H.Zaaijer@sanquin.nl (H.L.Z.); 2 Wageningen Bioveterinary Research, 8221 RA Lelystad, The Netherlands; wim.vanderpoel@wur.nl; 3 Academic Medical Center, 1105 AZ Amsterdam, The Netherlands

**Keywords:** hepatitis E, zoonotic infection, phylogenetic analysis

## Abstract

Pigs are suspected to be a major source of zoonotic hepatitis E virus (HEV) infection in industrialized countries, but the transmission route(s) from pigs to humans are ill-defined. Sequence comparison of HEV isolates from pigs with those from blood donors and patients in 372 samples collected in The Netherlands in 1998 and 1999 and between 2008 and 2015 showed that all sequences were genotype 3 except for six patients (with travel history). Subgenotype 3c (gt3c) was the most common subtype. While the proportion of gt3c increased significantly between 1998 and 2008, it remained constant between 2008 and 2015. Among the few circulating HEV subtypes, there was no difference observed between the human and the pig isolates. Hepatitis E viruses in humans are very likely to originate from pigs, but it is unclear why HEV gt3c has become the predominant subtype in The Netherlands.

## 1. Introduction

Until the beginning of this century, hepatitis E in the developed world was seen as a travelers’ disease and autochthonous HEV infection was rarely observed. More recently it became clear that indigenous infection with HEV genotype 3 is common in industrialized countries [1]. Five HEV genotypes are known to infect humans. Gt 1 and 2 only infect humans, while gt3 and gt4 infects humans and a wide range of animals (pigs, deer, wild boar, rabbits, mongoose) [2]. HEV genotype 7 circulates in camels and has occasionally been identified in humans [3]. Genotype 3 viruses are by far the most common in Europe. The distribution of nucleotide distances amongst HEV-3 subtypes shows a complex pattern with multiple hierarchies of relatedness. Therefore, a standard reference set of complete genome sequences has been proposed [4]. Genotype 3 viruses cluster in ten subtypes; a minority of sequences cannot be assigned to a subtype. The subtypes can be divided into three clades called 3abchij (which also contains 3k, 2l and 3m) and 3efg (also designated group 1 and group 2), and 3ra which is the subgenotype mostly found in rabbits [5,6]. The frequency at which the various subtypes occur varies by region and can change over time [7].

Domesticated swine form by far the largest known reservoir for HEV gt3 and gt4 and are considered the most likely source of human infections, although the transmission route(s) are not completely understood yet. In addition to consumption of undercooked meat products the virus may spread via surface water or crops; 17% of surface water samples in The Netherlands were HEV RNA positive in a 2009 study [8]. In The Netherlands more than 12 million pigs are reared and slaughtered each year [9]. More than 55% of pooled stool samples collected in pig farms tested HEV RNA positive in 2005 [10]. The incidence of HEV infection in The Netherlands is high compared to neighboring countries [11].

Approximately one percent of the Dutch blood donors seroconverted per year between 2009 and 2011, and one in 762 samples from blood donors tested HEV RNA positive in 2013 and 2014 [12,13]. For this study the results of surveillance of HEV sequences in Dutch pigs in 2000 and between 2008 and 2015 were combined with monitoring and sequencing of HEV from blood donors and HEV RNA positive patients. Comparisons and phylogenetic analyses of human and swine HEV sequences may help to understand transmission routes between pigs and humans.

## 2. Materials and Methods

### 2.1. Pig Samples

Swine fecal samples in The Netherlands were collected as part of an ongoing surveillance and characterization of bacterial agents. Between 2008 and 2015, around 150 individual fecal pig samples from different farms in The Netherlands were collected per year from pig herds delivered to the six largest slaughterhouses taking care of at least 60% of the total pig meat production in The Netherlands. All samples were collected during slaughter from the caecum or the rectum of fattening pigs of 5 to 6 months of age.

### 2.2. Human Samples

Human plasma samples between 2010 and 2015 were obtained from blood donors testing positive for HEV RNA during routine screening of selected plasma donors as described previously [13] and from HEV RNA positive patients that were submitted to Sanquin Diagnostic Services because of suspicion of (clinical) hepatitis E.

### 2.3. Molecular Detection of HEV

Swine fecal samples were suspended in PBS to a final dilution of 1:10 (*w*/*v*). The suspensions were mixed thoroughly and centrifuged in a tabletop centrifuge for 1 min at 13.000 g. 200 µL of supernatant was used to extract RNA with the High Pure RNA isolation kit (Roche, Mannheim, Germany). RNA was used immediately for HEV RT-PCR or stored at −70 °C until further testing. HEV detection by real-time RT-PCR was performed using undiluted RNA samples with the primers JVHEVF and JVHEVR as described by Jothikumar et al. [14]. HEV real time RT PCR was performed using the Invitrogen RNA UltraSence TM One-Step Quantitative RT PCR System Kit (Thermo Fisher, Waltham, MA, USA).

Human plasma samples were extracted using the QIAamp MinElute Virus Spin Kit (Qiagen, Hilden, Germany) using 400 µL of plasma. PCR was performed as published previously [13].

### 2.4. Nucleotide Sequencing of Pig Samples

A selection of at least 10 HEV PCR positive fecal swine samples (showing the lowest Ct-values), for each year was made to perform amplification and sequencing. Two fragments were amplified and sequenced from samples collected before 2010 as described previously, resulting in analysis of a 242 bp ORF1 and a 304 bp ORF2 fragment [15]. For pig samples collected between 2010 and 2015 a nested 493 bp ORF2 fragment that overlaps with the previously used 304 bp ORF2 fragment was amplified as described [16]. The PCR products were separated by electrophoresis on agarose gels and visualized under UV after ethidium bromide or Syber Safe staining. Positive RT-PCR products were sequenced in both directions. In total 69 ORF1 sequences and 99 ORF2 sequences were available from 142 fecal samples collected in 1998 and 1999 and between 2008 and 2015. This includes 32 sequences (17 ORF1 and 15 ORF2) from 19 fecal samples collected in 1998 and 1999 from one of our previous publications; these were included for comparison, and because no subtype analysis was performed in the past (Genbank accession numbers AF332620, AF335998-AF336014, AF336290-AF336299, AY032756-AY032759) [15].

### 2.5. Nucleotide Sequencing of Human Samples

Plasma samples from blood donors and patients were amplified using the same method as used for swine fecal samples from before 2010 [15]. Starting in 2015, a longer, overlapping, 1390 bp ORF2 fragment was amplified from samples with higher viral loads (>10,000 IU/mL) as published previously [6]. This longer sequence was available from 10 donors and patients. In total 217 ORF1sequences and 201 ORF2 sequences from 249 HEV isolates collected between 2010 and 2015 were available for analysis. This included 314 previously published HEV sequences that had not yet been subjected to subtype analysis (163 ORF1 and 151 ORF2; accession nr JX645320-JX645340, KM820620-KM820660, KR362770-KR362858) [5,12,17].

### 2.6. Phylogenetic Analysis

The HEV sequences from human and pig samples were aligned with the reference set as described by Smith et al. (2020) [4]. Phylogenetic trees of the aligned fragments were constructed using MegaX, applying neighbor joining as the tree building method, based on maximum composite likelihood distances, with 5000 bootstrap replications [18]. Positions with less than 75% site coverage were eliminated. Trees were visualized using FigTree v 1.4.2 (http://tree.bio.ed.ac.uk/software/figtree/ (accessed on 28 June 2021)) and further processed in Adobe Illustrator CS5. HEV subtypes were assigned using a tree containing all whole genome HEV sequences as previously published [4]. No subtypes were assigned to genotype 1 HEV sequences because of the limited number of gt1 infections. To reduce the number of branches, the phylogenetic trees shown in the figures were constructed using a selection of 51 HEV gt1–3 sequences with known genotype and subtype (if applicable). All sequences from the proposed reference set were included in this selection, plus two additional sequences for each subtype, if available [4]. These additional sequences were arbitrarily selected but based on the genetic distances (not outliers and no highly similar sequences were selected).

## 3. Results

The HEV subgenotype distribution in human and pig samples was determined by phylogenetic analysis from HEV sequences derived from samples collected between 1998 and 2015. Both ORF1 and ORF2 sequences were analyzed for this purpose. If both ORF1 and ORF2 sequences were available from the same sample, the longer ORF2 sequence was used for the final analysis. Concordance of results from ORF1 and ORF2 sequences was assessed in a separate analysis.

**Figure 1:**
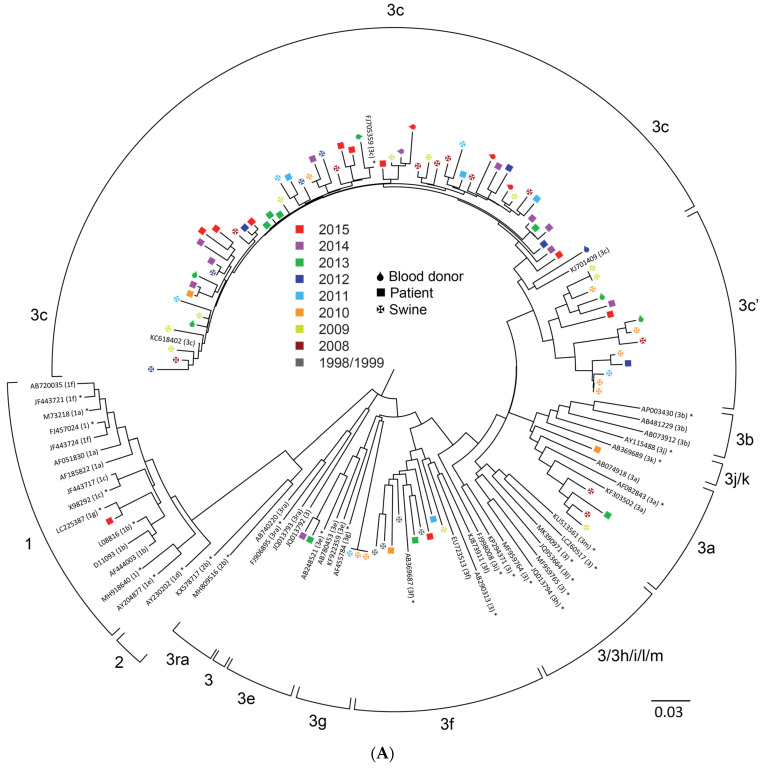

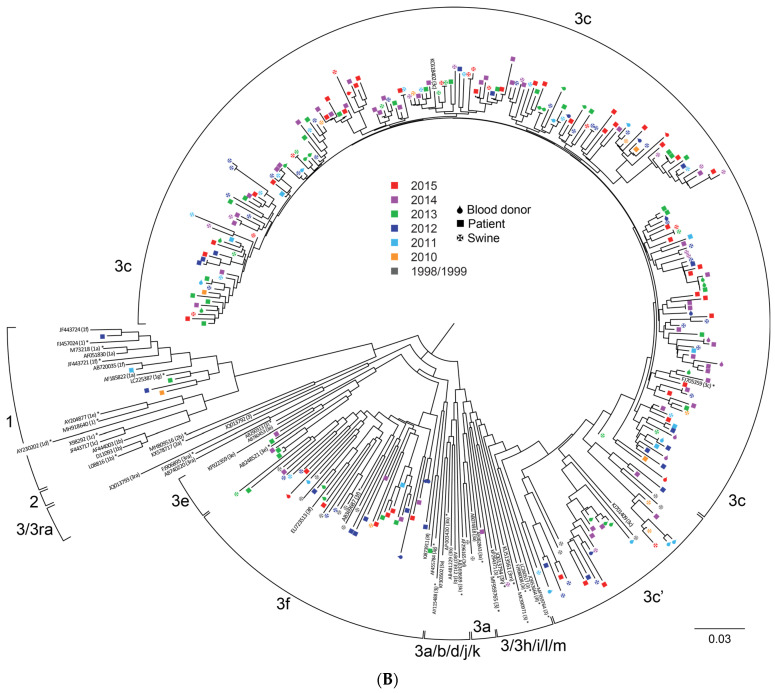
Phylogenetic trees of HEV ORF1 sequences (**A**) and HEV ORF2 sequences (**B**) from isolates from HEV RNA positive blood donors (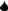), patients (■) and pigs (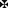). Phylogenetic analysis of the 242 bp ORF1 fragment and the 304 bp ORF2 fragment was performed in MEGA and visualized using FigTree. A selection of genotype 1–3 sequences with assigned (sub)genotype were used as reference sequences; the sequences from the proposed reference set are indicated with an asterisk (*) [4]. The circle around the tree indicates the subtypes; if no space was available subtypes were combined as indicated (e.g., 3h/i). The scale bar at the bottom right denotes evolutionary distance.

**Figure 2:**
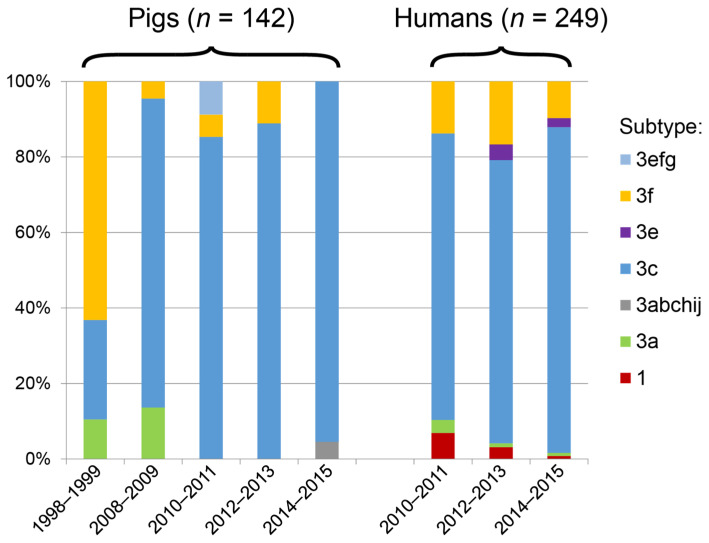
Summary of the difference in subgenotype distribution of HEV isolated from pigs and humans over time. The percentage of HEV sequences from each subtype from pig and human HEV was calculated from Table 1.

In total, the subtypes from 91 HEV ORF1 sequences and 300 HEV ORF2 sequences was determined in samples from 142 pigs, 196 patients and 53 blood donors (Table 1). Phylogenetic trees are shown in Figure 1, and Table 1 lists the number of isolates (ORF1 and ORF2 results combined) of each subtype in the various years. Results from Table 1 are summarized in Figure 2. Details about all samples and sequences can be found in Supplementary Tables S1 and S2.

In the phylogenetic analysis no clustering of human or swine HEV sequences was observed by visual inspection of the trees, with the exception of a relatively large number of gt3f swine samples from 2008 and 2009 in a region of the tree where relatively few human sequences are located. This was confirmed by an analysis of the subtype distribution (Table 1) that showed that the proportions of the different subtypes during the period 2008–2015 was similar in humans and pigs but changed between 1998/1999 and 2008. Using a Chi square test, the difference was only significant between humans and swine sequences for genotype 3efg (observed in swine samples three times and not in humans; *p* = 0.02). As expected, genotype 1 was only found in human samples.

Genotype 3 sequences cluster in two clades (3abchij and 3efg). The percentage of group 3abchij sequences relative to the total number of genotype 3 sequences in pigs, blood donors and patients is shown in Figure 2. Overall, 84% of the sequences belonged to the 3abchij group (83.8% in swine samples and 84.0% in human HEV isolates). While the proportion of 3abchij isolates did not differ significantly between human and pig isolates, the proportion of 3abchij isolates in swine increased significantly from 37% in 1999 and 2008 to 91% in the period 2008–2015 (*p* < 0.0001). Unfortunately, no isolates from blood donors or patients were available from this period.

The consistency of the result was checked by comparing the assigned ORF1 and ORF2 subtypes from the samples of which both ORF1 and ORF2 sequences were known. The subtypes were identical in 192 of the 194 isolates (99%; data not shown). In one case no subtype could be assigned for the ORF1 sequence (i.e., 3efg) while the ORF2 sequence was gt3f. The sequences therefore belonged to the same clade. In one case a peculiar discrepancy was found (ORF1: 3c, ORF2: 3f) in which the sequences belonged to different clades. In conclusion, the ORF1 and ORF2 based subtype assignment was almost always identical, and both fragments can be used for reliably assigning subtypes.

Genotype 3c sequences can be divided in two groups (albeit somewhat arbitrary), depicted as 3c and 3c’ in Figure 1. Approximately 14% of all 3c HEV sequences are in the 3c’ cluster. Bootstrap support for the division of 3c sequences is limited (82% for ORF1 and non-significant for ORF2 sequences). However, the assignment of HEV 3c’ was consistent for all 22 HEV isolates from which both ORF1 and ORF2 sequences were available, suggesting that the sequence from this group consistently differs throughout the genome.

## 4. Discussion

In our study a comparison was made of the HEV (sub)genotype distribution of human and swine HEV sequences. There were no significant differences in the subtype distribution between HEV sequences from human and swine, which is compatible with the assumption that HEVs from swine are the major source of HEV infections in humans. For the subtyping and comparison of humans and swine populations the analysis based on partial sequences should be sufficient. For individual analysis whole genome would be preferred, however this was not the aim for this study. Whole genome sequencing could also be used to confirm the suggestion that genotype 3c can be divided in two groups (tentatively called 3c and 3c’ here).

Domestic pigs form by far the largest reservoir for gt3 HEV in Europe, as the number of animals of other species in which HEV gt3 sequences highly homologous to the ones infecting humans have been detected (wild boar, deer and mongoose) is relatively small and the prevalence of HEV infection among pigs is high [2,10]. Direct transmission, environmental transmission and consumption of undercooked pig meat and meat products may be routes of HEV genotype 3 infections in humans. The seroprevalence in swine herds is very high in many countries. In an increasing number of studies, the consumption of pig meat or meat products has been identified as a risk factor for HEV infection [19,20,21].

In The Netherlands, HEV specific antibodies were detected in ~70% of slaughter pigs already in 2004 [22]. 27% of pork liver and meat products were HEV RNA positive in 2016 suggesting many pigs are viremic at the time of slaughter [23]. Seroprevalence in wild boar in The Netherlands in 2016 was around 20%, which is lower than in domestic pigs (unpublished observation, W. van der Poel). To this date only genotype 3 HEV has been detected in pigs, including wild boar, in The Netherlands.

The number of HEV gt3 infections in humans in The Netherlands is relatively high, with an estimated incidence of 1.1% between 2009 and 2011 [12]. Screening of a selection of plasma donations in pools of 96 donations showed that in 2013 and 2014 one in 1322 pools was HEV RNA positive and one in 762 donations was positive when tested individually [13]. In similar studies in other European countries most of the reported incidence rates were lower (1:8416 in Austria [24], 1:2448 in England [25], 1:2330 in Denmark [26]; in Germany rates of 1:840 and 1:1240 have been reported [21,27]). HEV RNA has been detected in Dutch pig livers from butcher shops as well as in various porcine pooled blood products [28,29], showing that a significant number of animals is HEV RNA positive at the moment of slaughter.

We observed no difference in HEV genotype distribution between humans and pigs, which indicates that pigs are a likely source of HEV infections in humans in The Netherlands. In contrast, a significant difference was reported in the UK where 96% of HEV RNA positive pig samples from 2013 that could be genotyped fell within the genotype 3efg clade, while only 32% of the patients and blood donors with HEV infection between 2012 and 2014 was infected with group 3efg HEV [7,20]. Even though the pig samples were all collected in one year, the different HEV subtypes observed in humans and pigs may reflect the fact that the majority of pork for consumption in the UK is imported from mainland Europe. The difference in subtypes may be a relatively recent phenomenon: between 2009 and 2012 a strong increase in the number of HEV infections was reported in England and Wales, concomitant with a gradual strong increase in the frequency of infections with clade 3abchij HEV strains in human patients. During this period the frequency of infection with clade 3abchij isolates in British HEV patients increased from less than 10% to 58%. Our data show that a strong shift in the ratio of 3efg to 3c isolates in pigs also occurred in The Netherlands between 2000 and 2008 (Figure 2); the percentage of 3c sequences increased from 26% to 82% in this period. Even though the number of known HEV sequences from this period is limited, 35 HEV sequences from patients are available from the period 2002–2006 in the literature [30]. Phylogenetic analysis of these sequences using the current reference set showed that 52% of the 31 genotype 3 sequences were genotype 3c (data not shown). In addition, a Genbank search for HEV sequences originating from The Netherlands resulted in identification of 38 unpublished pig sequences isolated in 2005 from pig stool and surface water (EU526606-526647; submitted by the Dutch National Institute for Public Health and the environment). 15 of these sequences (39%) were genotype 3c (data not shown). Thus, a re-analysis of available Dutch HEV sequences from other labs from the period 2002–2008 confirms that the proportion of genotype 3c infections steadily increased between 2000 and 2008, both in pigs and in patients.

Data from France suggest that a slower and less pronounced transition from HEV type 3efg to 3abchij infections is also taking place in France. The frequency of genotype 3f infections in the Midi-Pyrénées area gradually decreased from 90% to 65% in the period 2003–2014 while the number of HEV gt 3c infections increased from 5 to 24.5% [31]. The percentage of 3c infections in the remaining part of France was 20% between 2012 and 2014 [31], and increased to 26.7% in 2015 and 2016 suggesting a transition from 3efg to 3abchij strains may still be continuing [32].

Our data indicate that HEVs originating from pigs are a likely source of HEV infections in the general population in The Netherlands. It is less clear what the main transmission routes are. These may include the consumption of insufficiently heated pork liver, liver products or meat products, crops that have been irrigated with surface water and food products containing porcine blood products such as spray-dried plasma, hemoglobin and fibrinogen [1,29,33]. Considering the relatively high incidence of HEV infection in The Netherlands, measures to reduce circulation of this human pathogen in commercial pig farms need to be considered; in particular interventions that reduce the number of HEV positive animals at the time of slaughter. While raising pigs on farms free of HEV may be preferable in the long run, vaccination strategies may be an alternative way to reduce the amount of virus entering the food chain, however until this date there is no commercial vaccine available for pigs. A vaccine against HEV is registered in China however it has not yet been approved in other countries [34]. Virus introduction routes in pig farms have not been completely clarified but intervention strategies will certainly involve changes in farm management practices. To keep pig farms free of HEVs biosecurity will have to be enhanced and the need to develop and apply vaccines may increase. Finally, strict surveillance and monitoring of HEV infection in pigs may have to be implemented.

## Figures and Tables

**Table 1:** Distribution of HEV sequences among the various HEV subtypes over the years in pigs and humans. The total number of all subtypes as depicted in Figure 1 was calculated (ORF1 and ORF2 combined). If no subtype could be assigned the sequence were classified as clade 3efg or 3abchij. For genotype 1 no subtypes were assigned because of the low number of gt1 infections.

Pig Sequences	Year: 1998–1999	2008	2009	2010	2011	2012	2013	2014	2015	Total
3a	2	2	1							5
3c	5	9	9	12	17	31	9	11	10	113
3abchij								1		1
3f	12		1	1	1	4	1			20
3efg				2	1					3
Total	19	11	11	15	19	35	10	12	10	142
**Human** **Sequences**	**Year:** **1998–1999**	**2008**	**2009**	**2010**	**2011**	**2012**	**2013**	**2014**	**2015**	**Total**
1				1	1	2	1		1	6
3a				1			1	1		3
3c				4	18	24	48	57	50	201
3e							4	3		7
3f				1	3	9	7	4	8	32
Total	0	0	0	7	22	35	61	65	59	249

## Data Availability

Not applicable.

## Supplementary Materials

The following are available online at https://www.mdpi.com/article/10.3390/v13071265/s1, Figure S1: Phylogenetic trees of HEV ORF1 sequences (a) and HEV ORF2 sequences (b) from isolates from HEV RNA positive blood donors (), patients (■) and pigs (). Phylogenetic analysis of the 242 bp ORF1 fragment and the 304 bp ORF2 fragment was performed in MEGA and visualized using FigTree. A selection of genotype 1–3 sequences with assigned (sub)genotype were used as reference sequences; the sequences from the proposed reference set are indicated with an asterisk [4]. The circle around the tree indicates the subtypes; if no space was available subtypes were combined as indicated (e.g., 3h/i). Figure S2: Summary of the difference in subgenotype distribution of HEV isolated from pigs and humans over time. The percentage of HEV sequences from each subtype from pig and human HEV was calculated from Table 1. Table S1 and Table S2: overview of the ORF1 and OEF2 sequences used for the phylogenetic analysis.
